# Behavioral differences following ingestion of large meals and consequences for management of a harmful invasive snake: A field experiment

**DOI:** 10.1002/ece3.4480

**Published:** 2018-09-05

**Authors:** Shane R. Siers, Amy A. Yackel Adams, Robert N. Reed

**Affiliations:** ^1^ USDA APHIS WS National Wildlife Research Center Hilo Hawaii; ^2^ US Geological Survey Fort Collins Science Center Fort Collins Colorado

**Keywords:** automated telemetry, behavioral ecology, brown treesnake (*Boiga irregularis*), detectability, foraging ecology, submergent behavior

## Abstract

Many snakes are uniquely adapted to ingest large prey at infrequent intervals. Digestion of large prey is metabolically and aerobically costly, and large prey boluses can impair snake locomotion, increasing vulnerability to predation. Cessation of foraging and use of refugia with microclimates facilitating digestion are expected to be strategies employed by free‐ranging snakes to cope with the demands of digestion while minimizing risk of predation. However, empirical observations of such submergent behavior from field experiments are limited. The brown treesnake (Serpentes: Colubridae: *Boiga irregularis*) is a nocturnal, arboreal, colubrid snake that was accidentally introduced to the island of Guam, with ecologically and economically costly consequences. Because tools for brown treesnake damage prevention generally rely on snakes being visible or responding to lures or baits while foraging, cessation of foraging activities after feeding would complicate management. We sought to characterize differences in brown treesnake activity, movement, habitat use, and detectability following feeding of large meals (rodents 33% of the snake's unfed body mass) via radio telemetry, trapping, and visual surveys. Compared to unfed snakes, snakes in the feeding treatment group showed drastic decreases in hourly and nightly activity rates, differences in refuge height and microhabitat type, and a marked decrease in detectability by trapping and visual surveys. Depression of activity lasted approximately 5–7 days, a period that corresponds to previous studies of brown treesnake digestion and cycles of detectability. Our results indicate that management strategies for invasive brown treesnakes need to account for cycles of unavailability and underscore the importance of preventing spread of brown treesnakes to new environments where large prey are abundant and periods of cryptic behavior are likely to be frequent. Characterization of postfeeding behavior changes provides a richer understanding of snake ecology and foraging models for species that consume large prey.

## 1 INTRODUCTION

Empirical documentation of the postfeeding behaviors of snakes from field experiments is extremely sparse. We know of only two such studies: desert rattlesnakes (*Crotalus* spp.; Beck, 1996) and a temperate forest colubrid (*Elaphe obsoleta*; Blouin‐Demers & Weatherhead, 2001). Both studies focused on thermoregulation of primarily terrestrial snakes under broad daily temperature fluctuations. The brown treesnake (*Boiga irregularis*) is a tropical, nocturnal, arboreal colubrid and a notorious invasive alien species on the island of Guam. In this study, we sought to evaluate the behavioral changes exhibited by brown treesnakes following ingestion of large prey items and to interpret our observations in the contexts of behavioral ecology and invasive species management.

As is true of most predators, foraging behaviors of predatory reptiles are typically shaped by external factors (prey availability, predation risk, social interactions, habitat structure, and opportunities for thermoregulation), internal factors (hunger, experience, age, size, sex, and reproductive state), and physiological factors (sensory, morphological, and behavioral characteristics) (after Perry & Pianka, 1997; Vitt & Pianka, 2007). The frequency, duration, and distance of foraging movements are associated with two broadly recognized foraging modes of reptiles. Some reptiles are sit‐and‐wait predators, or ambush predators, that invest little time and energy into searching for prey, remaining stationary and attacking mobile prey, and tend to be characterized by a short and stout body form. Others are active foragers, or wide foragers, which move through the environment in search of mobile or nonmobile prey, and have a longer and narrower body form with higher energetic demands and metabolic rates (e.g., Secor & Nagy, 1994). As with most dichotomies, this is actually a spectrum with most species exhibiting both modes in varying proportions (Perry & Pianka, 1997).

Snakes are the only terrestrial vertebrates that specialize in swallowing large prey whole, and this has implications for their anatomy, physiology, and behavior. Consuming large meals was made possible through evolutionary modifications of the skull and specializations associated with methods of detecting, capturing, subduing, swallowing, and digesting prey, and may have enabled the successful radiation of snakes (Cundall & Greene, 2000; Gans, 1961; Greene, 1983; Pough, 1983; Shine, 1986). Some snakes routinely ingest meals 20%–60% of their own body mass, and a few can take meals of even greater mass than their own (Greene, 1983, 1992, 1997; Secor & Diamond, 1998).

The drastic increase in mass and radical alteration of the slender body form of a snake caused by a large prey bolus may result in distinct postprandial behavioral changes. We propose that the nature and magnitude of such changes are driven by the physiological demands associated with digestion, the need to avoid predation while handicapped by the gastrointestinal prey burden, or both. These demands are likely to result in “submergent behavior” (Maiorana, 1976) upon finding a resting site that provides the appropriate microclimate for digestion and shelter from predators.

### 1.1 Metabolic and cardiovascular demands of digestion

Beyond the energetic costs of capturing, handling, and swallowing prey, the metabolic demands of digestion of large prey are considerable. The collection of physiological processes and increased metabolic expenditures that occur in postprandial animals is referred to as “specific dynamic action” (SDA; reviewed in McCue, 2006). These processes are complex and not fully understood, but include protein and hormone production, secretion of digestive acids and enzymes, alkalinization of blood, and increases in the mass and function of the intestines, heart, pancreas, liver, and kidneys. This rapid phenotypic change places extensive demands on metabolic activity. Postprandial metabolism and oxygen consumption can surpass that attained during exercise (Cruz‐Neto, Andrade, & Abe, 1999; Overgaard, Busk, Hicks, Jensen, & Wang, 1999; Secor & Diamond, 1998). Oxygen consumption, digestion time, kidney hypertrophy, amino acid uptake rates, etc., can increase with prey size (Cruz‐Neto et al., 1999; Secor & Diamond, 1997).

Exploiting very large prey also necessitates that digestion is efficient over a short period of time, to reduce the probability of putrefaction of the prey (Cundall & Greene, 2000). Digestion in ectothermic animals is highly temperature‐dependent, such that recently fed snakes must seek appropriate thermal microclimates for efficient digestion (Beck, 1996; Blouin‐Demers & Weatherhead, 2001; Peterson, Gibson, & Dorcas, 1993). Further, the anatomical position of the stomach ventral to, and overlapping with, the lungs (or a part of the lungs) can impact ventilation itself. Consumption of large meals may reduce tidal volume and temporarily reduce vital capacity and maybe even blood flow, as suggested by Rosenberg (1973) and Secor (2008).

### 1.2 Vulnerability to predation

Organisms need to balance foraging and feeding with predator avoidance, and the risk of predation is important in altering behavior of foragers (Sih, 1980). Taking large prey increases predation risk for snakes at the outset, because the snake may be incapacitated while subduing and ingesting prey, which may take an extended period of time, and prey handling time increases with prey size (Cruz‐Neto et al., 1999; Nielsen, Jacobsen, & Wang, 2011). Snakes typically avoid predation by crypsis, flight, and defensive behaviors.

#### 1.2.1 Crypsis

Camouflage, immobility, sheltering in refugia, or a combination, thereof, is typically the lowest cost and most fundamental set of predator avoidance behaviors. Foraging, particularly active foraging, puts snakes at a heightened risk of predation by foregoing crypsis and compelling movement away from shelter. Upon meeting its immediate feeding requirements by the acquisition of a large prey item, a foraging snake can decrease its vulnerability to predation by simply ceasing to forage and to again avail itself of the defenses of crypsis and shelter. Reduction in foraging behavior to avoid predation has been termed “submergent behavior” (Maiorana, 1976).

#### 1.2.2 Flight

Addition of mass to an animal may be expected to influence its locomotory behavior and capabilities (Coombs, 1978; Taylor, Heglund, McMahon, & Looney, 1980). The weight and bulk of a meal in the gut of a snake are much more directly coupled to the mechanics and movement of the propulsive structures of snakes, compared to limbed animals; ingesting large meals changes the mass and shape of the animal, imposing locomotor constraints (Crotty & Jayne, 2015). Predator avoidance via rapid flight can be seriously impaired due to these locomotor hindrances. In the laboratory experiments, feeding of meals up to 50% relative prey mass (hereafter, “RPM”) has resulted in decreases in sprint speed, average speed, and endurance in juvenile gartersnakes (*Thamnophis elegans* and *T. marcianus*) and trinket snakes (*Elaphe helena*) in response to simulated predator attacks (Ford & Shuttlesworth, 1986; Garland & Arnold, 1983; Mehta, 2005). While a large prey bolus may not alter the adaptive advantage of dorsal pigmentation patterns associated with crypsis or defense, striped and unicolored‐speckled patterns are associated with antipredator strategies emphasizing flight (Jackson, Ingram, & Campbell, 1976). Impediments to locomotion imposed by burdensome gut contents may negate the adaptive advantage of these patterns of coloration. Ford & Shuttlesworth (*ibid*.) noted that in some trials the interference of prey stiffness with lateral undulation exceeded the effect of actual mass ingested. In addition to the aerobic demands of lugging a greatly increased mass, the metabolic demands of digestion can further decrease aerobic scope and endurance (Crotty & Jayne, 2015). Secor and White (2010) demonstrated that, when faced with the dual cardiovascular demands of digestion and flight, blood flow in Burmese pythons (*Python bivittatus*) fed rodent meals equaling 24.7% of the snake's body mass was diverted from the viscera to the axial muscles for escape behavior.

#### 1.2.3 Defensive behaviors

Appropriate antipredator behavior may be contingent on internal factors that affect flight speed and endurance (Herzog & Bailey, 1987), and organisms may modify their behavior to compensate for morphological changes (Shine, 1980). While capacity for flight is limited by the burden of a large prey mass, other defensive behaviors may be invoked when a snake encounters a predator. Herzog & Baily (*ibid*.) reported that 10‐week‐old gartersnakes (*T. sirtalis*) fed four hours previously were more likely to strike a threatening stimulus (human hand) than to flee as unfed snakes did. Mehta (2005) observed that hatchling *E. helena* that had consumed 50% or more RPM did not flee, but rather assumed nonthreatening cryptic antipredator postures when predation was simulated, while snakes fed 0%–35% RPM exhibited active or threatening responses.

Metabolic and cardiovascular demand, locomotor impairment, and predation risk effects of large meals may be even more consequential for arboreal snakes that typically have an attenuated body form as an adaptation for arboreal locomotion (Feldman & Meiri, 2013; Lillywhite & Henderson, 1993; Pizzatto, Almeida‐Santos, & Shine, 2007). Meals that alter the mass and balance of arboreal snakes alter the match between the mass of the snake and the strength and stiffness of supporting perches; cantilever abilities required to negotiate gaps are likely to be extremely reduced, and the effect of prey stiffness on axial bending presents a more acute problem for slender snakes (Crotty & Jayne, 2015).

### 1.3 The brown treesnake

**Figure 1:**
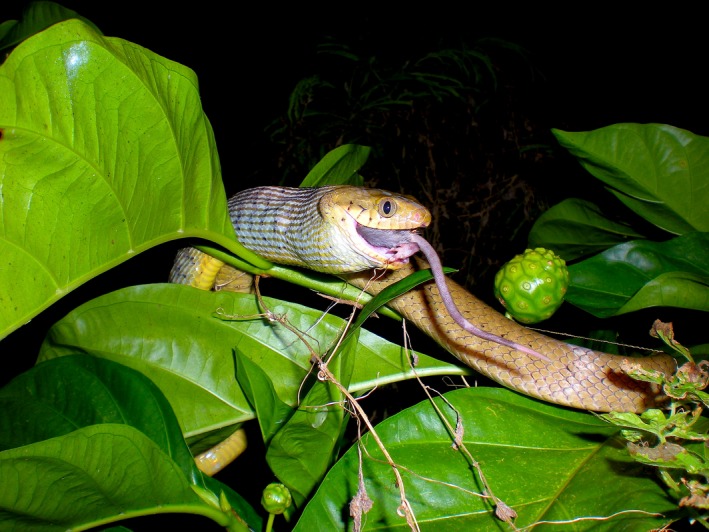
Brown treesnake ingesting a rodent meal (photograph by Michael Hogan, taken during a separate study)

The brown treesnake (*Boiga irregularis*; Figure 1) is an arboreal, nocturnal, oviparous, mildly venomous, rear‐fanged colubrid snake that is physiologically similar to other active foraging arboreal snakes (as characterized in Lillywhite & Henderson, 1993), with an attenuated and light weight body form and cryptic countershaded coloration (brown to olive dorsum and white to yellow venter). They are mixed‐strategy foragers, switching between sit‐and‐wait and active foraging modes within the same night, feeding on both active and inactive prey (Rodda, 1992).

Native to Papua New Guinea, the Solomon Islands, Indonesia, and northern and eastern coastal Australia, the brown treesnake has gained notoriety for catastrophic ecological effects and significant economic burdens following its accidental introduction to the island of Guam during, or shortly after, World War II (Rodda, Fritts, McCoid, & Campbell, 1999; Rodda & Savidge, 2007). From a presumably very small number of founders (Richmond, Wood, Stanford, & Fisher, 2015), the invasion front engulfed all of Guam's terrestrial habitats by the 1980s and reached population densities in excess of 50 snakes per hectare (Rodda, McCoid, Fritts, & Campbell, 1999; Savidge, 1987). Results of this invasion included the extirpation or extinction of nearly the entire native forest avifauna (Savidge, 1987; Wiles, Bart, Beck, & Aguon, 2003), with cascading ecological and economic consequences (e.g., Perry & Morton, 1999; Rogers, Hille Ris Lambers, Miller, & Tewksbury, 2012; Rogers et al., 2017), and impacts on domestic poultry production, tourism, and human health (Fritts & McCoid, 1991; Fritts, McCoid, & Haddock, 1990; Rodda & Savidge, 2007). Because Guam is a major hub for commercial and household goods throughout the Pacific, economic impacts are increased by the cost of developing, testing, and implementing tools and strategies to reduce brown treesnake abundance and preventing accidental transportation of brown treesnakes to other Pacific islands such as Saipan, Rota, Tinian, and Hawai'i (Clark, Clark, & Siers, 2018; Engeman & Vice, 2001; Pimentel, Lach, Zuniga, & Morrison, 2000).

Much of the damage caused by brown treesnakes stems from their climbing and feeding behaviors. They shift between various modes of locomotion depending on the nature of the substrate (Chiszar, 1990) and have exceptional gap‐bridging abilities (Jayne, Lehmkuhl, & Riley, 2014). Their inclination to forage on power transmission lines has led to power outages, estimated to have cost in excess of $4.5 M per year over a 7‐year period (Fritts, 2002).

The brown treesnake is a generalist predator that consumes all life stages of vertebrate prey including eggs, juveniles, and adults (Rodda, Fritts, et al., 1999) and kills by constriction as well as envenomation (Rochelle & Kardong, 1993; Shine & Schwaner, 1985). Brown treesnakes undergo an ontogenetic prey shift from mostly lizards as juveniles to a preference for endothermic prey (birds and mammals) as adults (Greene, 1989; Lardner, Savidge, Rodda, & Reed, 2009; Savidge, 1988; Shine, 1991; Siers, 2015), a common pattern for arboreal snakes (Lillywhite & Henderson, 1993). Although hatchling and juvenile brown treesnakes are almost exclusively arboreal, on Guam, adults shift toward more terrestrial movement and foraging (Rodda, 1992; Rodda & Reed, 2007; Siers, 2015).

Most brown treesnake prey items are relatively small, but some may weigh up to 60% of the snake's mass (Chiszar, 1990). The brown treesnake is flexible in predatory behavior, ability to subdue large prey by constriction, and ability to swallow large prey in an energetically efficient manner. These characteristics enable it to exploit a prey base containing species that vary greatly in size and habits, which likely contributed to its success as a colonizer (Chiszar, 1990; Rodda, 1992). The brown treesnake attains an unusually large size for an arboreal colubrid (total lengths up to 2.3 m for females and 3.1 m for males; Rodda, Fritts, et al., 1999), increasing the size range of prey that might be attacked and swallowed.

The effectiveness of various tools for the capture or lethal control of invasive brown treesnakes depends largely on detectability or targetability; that is, given a quantified level of effort, what is the probability of detecting, capturing, or killing an individual snake within the activity area? Effectiveness of visual surveys, trapping, and toxic bait tools for brown treesnake control has been demonstrated to be influenced by internal factors (sex, size, and body condition) and external factors (availability of alternative prey; Gragg et al., 2007; Rodda, Savidge, Tyrrell, Christy, & Ellingson, 2007; Tyrell et al., 2009; Christy, Yackel Adams, Rodda, Savidge, & Tyrrell, 2010; Lardner et al., 2013; Christy, Savidge, Yackel Adams, Gragg, & Rodda, 2017; Siers, Savidge, & Reed, 2017). These factors have also been indicated to influence brown treesnake movement characteristics (Santana‐Bendix, 1984; Tobin, Sugihara, Pochop, & Linnell, 1999; Siers, Reed, & Savidge, 2016; Christy et al., 2017). If brown treesnakes decrease movement and foraging during digestion, this will have implications for the effectiveness of various control tools that typically rely either on visual detection by human searchers or response of foraging snakes to lures or baits (e.g., Christy et al., 2010; Clark et al., 2018; Engeman & Vice, 2001; Lardner et al., 2013; Tyrell et al., 2009).

### 1.4 Motivation and hypotheses

Cycles of foraging and prolonged refuge have been reported with observational data from natural and seminatural environments (e.g., Luiselli & Agrimi, 1991; Phelps, 2002; Saint‐Girons, 1979); however, little work has been dedicated to assessing the effects of large meals on postfeeding behavior of snakes in field experiments. In addition to increasing our understanding of snake foraging behavior, knowledge of invasive brown treesnake activity is important for predicting the effectiveness of control programs. We hypothesized that brown treesnakes that had recently ingested large prey items would exhibit reduced movement behavior and that such reductions would reduce the effectiveness of invasive species control tools that rely in part on snake movement. In particular, we predicted that, compared to unfed snakes, snakes fed a relatively large meal would (a) exhibit reduced hourly activity patterns, (b) make shorter daily movements, (c) select different daytime resting locations, and (d) be less detectable/targetable by management tools.

## 2 METHODS

The general study design was to monitor the behavior of snakes that had been feed a standardized large meal, proportional to their body mass, and make direct comparisons to a control group of snakes that had not been fed a meal.

### 2.1 Study site

The experiment took place in the U.S. Geological Survey's brown treesnake study enclosure on Northwest Field of Andersen Air Force Base, Guam. This 5‐ha parcel of limestone forest and secondary forest is surrounded by a two‐way snake barrier, comprised of a chain‐link fence sheathed on both sides with ¼” (6.35 mm) galvanized wire mesh. The mesh on both sides is formed with an approximately 10–12 cm diameter “bulge” at 1.2 m above ground level; snakes attempting to scale the vertical mesh lose purchase when attempting to navigate the bulge. This site was selected for the study for several reasons: (a) The habitat is representative of much of Guam's forest habitats and allows the full behavioral repertoire of brown treesnakes during every phase of their life cycle; (b) the barrier restricts study snakes to a range within which radio telemetry signals could be reliably received and recorded by a stationary data logger; (c) snakes within the population were enumerated and individually identified with PIT tags, with running histories of captures and vital rates; and (d) a concurrent trapping and visual survey study allowed the evaluation of effects of recent feeding on detectability with these typical survey and control techniques. Based on capture records for the preceding year, we estimate the population of snakes in this enclosure at 125 individuals (25 per ha) during the time of the study, a relatively typical density for forest habitat on Guam (Rodda, McCoid, et al., 1999).

### 2.2 Feeding treatment

At the beginning of each trial, snakes within the barrier were captured by trapping (double‐funnel wire mesh trap; Rodda, Fritts, Clark, Gotte, & Chiszar, 1999; Tyrell et al., 2009) or visual detection and hand capture (using gloves, snake hooks, and tongs as necessary; Christy et al., 2010). Upon capture, current morphometric information was recorded: Snout‐vent length (SVL; mm) was measured by gently stretching the snake straight along a flexible cloth tape; weight (g) was measured with Pesola spring scales (Pesola AG, Schindellegi, Switzerland); and sex was determined by probing for retracted hemipenes (Reed & Tucker, 2012). Because probing is prone to error, particularly for small snakes, we assigned sex based on consensus from multiple repeated sexing attempts during and prior to this study. Snakes that were molting, apparently gravid, or exhibiting an obvious prey bulge were not selected for the study. Snakes were temporarily held at the location of capture in breathable cloth snake bags until assigned to a treatment group (1–14 hr). Snakes assigned to the feeding treatment group were then placed in a feeding chamber comprised of a ventilated 10‐gallon plastic storage tub furnished with a textured rubber floor (for locomotor purchase and ease of sanitation) and a length of PVC tube cut longitudinally to provide a refuge. Feeding chambers were positioned at or near the site of capture, with minor adjustment of locations to avoid direct sunlight. Upon determination of snake weight, a preweighed frozen mouse or rat (RodentPro, Inglefield, Indiana) was selected to match 33% of the snake's prefed body mass (roughly equivalent to the meal size identified by Collins and Rodda (1994) as being reliably ingested without risk of asphyxiation). A 2.8‐g tip‐sensitive VHF radio transmitter (Model PD‐2P, Holohil Systems Ltd., Carp, Ontario) was implanted in the rodent's body cavity. External wire transmitter antennae were trimmed to a length of approximately 100 mm; such transmitters have been previously demonstrated to pass from brown treesnake GI tracts through defecation or regurgitation without problem (Siers et al., 2016). The snake and rodent were placed in the feeding chamber, and the snake left to voluntarily consume the rodent meal; if the rodent was not consumed by the end of the day's field activities, the snake was left in the feeding chamber overnight. Once the rodent was ingested, the snake was gently tipped from the feeding chamber and left to naturally seek a refuge. If a snake refused to ingest the offered rodent for 24–36 hr, it was either released or assigned to the unfed control group, depending on the sampling requirements at the time. Snakes assigned to the unfed control group were fed a transmitter which was manually massaged down the esophagus into the stomach, aided by application of a water‐based lubricant. Force feeding of transmitters alone is a lower stress procedure relative to surgical implantation and has been successfully employed with no detected behavioral artefacts in a previous study (Siers et al., 2016). A total of 62 snakes were used in this study. The maximum number of trials per snake was 6 (2 snakes); most repeated trials were transmitter‐only treatments, which were repeated more frequently to increase sample size due to more rapid gut passage. Thirty‐four snakes received feeding treatments; most (24 snakes) were fed only once, and no snake was fed more than three times (2 snakes).

Initial treatment group assignments were random. Subsequent assignments were made to ensure that snake sex ratio and size distributions matched between treatment groups, and snakes previously assigned to one treatment group were assigned to the other group when used in the study more than once. Snakes in both study groups were left to naturally pass transmitters through defecation or regurgitation. We sought to balance sample sizes between treatment groups; however, snakes fed transmitters alone tended to pass them more quickly, so more snakes were eventually assigned to the control group to ensure a balance of observation days.

### 2.3 Statistical analysis

Statistical models were linear or generalized linear mixed‐effects models with response variable distribution families (normal, logistic, or negative binomial) as applicable. Where appropriate, statistical models considered or controlled for the influence of individual snake characteristics: sex [SEX], size (snout‐vent length [SVL] and its quadratic form [SVL^2^]), and body condition (with condition index [CI] being the residuals from a fourth‐order polynomial regression of the natural log of body mass against the natural log of SVL); these were modeled as fixed effects. All models determining the significance of effects included a random effect of individual snake identification code [ID] to account for multiple repeated measures on individuals. The fixed effect of primary interest was the feeding status of the snake [MEAL], a categorical term indicating whether the snake had or had not been fed a large meal at the outset of the trial. Additional terms were added to models as pertinent to the particular inquiry. Possible combinations of model terms were considered in an AIC_*c*_ multimodel inference framework (Burnham & Anderson, 2002), with sums of weights of all models containing a particular (*i*th) term (*Σw*_*i*_) interpreted as relative variable importance. Model sets were constrained to include all lower order main effects (when evaluating quadratic or interaction terms) and other terms structurally implicit to the model (i.e., random effect and autoregression terms). Models within 2 AIC_*c*_ units of the top model were considered plausible alternative models. Significance of the main effect [MEAL] was also investigated by a likelihood ratio test (ANOVA comparison of models with and without the term). Because standard methods have not been developed for predictions from models containing random terms, analogous fixed‐effects models were used to evaluate and plot effect sizes; however, all reported *p*‐values and AIC_*c*_ values are based on mixed‐effects model results. Statistical significance was set at *α* = 0.05. Time intervals are reported rounded to the whole hour for simplicity; for example, a reported time interval of “1800–1900,” or simply “1800,” actually represents the interval 1800.000 to 1859.999.

### 2.4 Real‐time logging of hourly activity patterns

Frequency of small‐scale movements was continually monitored through transmitter pulses recorded by a TR‐5 Telemetry Scanning Receiver and data acquisition system (Telonics, Inc., Mesa, Arizona). Transmitters were equipped with a two‐position switch that changes pulse rate when the body position of the animal changes more than 10° from the switch's preset orientation. Changes in pulse rate serve as a proxy measurement for snake activity, under the logic that a relatively stationary/sedentary snake would cause the transmitter to tip less frequently that an actively moving/foraging snake would.

Transmitter pulse data were recorded continually for 7 days following the release of each snake, except during periods when the receiver was being reprogrammed to add or delete frequencies. Because field activities, which generally occurred between 1000 and 1200 hr on each day, could have influenced the activity of resting nocturnal snakes, data are only reported from 1200 each day until 1000 the following day (22 hr per day). Some field activities occurred outside of these hours, but such activities were conducted so as to minimize disturbance to snakes carrying transmitters and reduce unintentional effects on movement data. For example, technicians conducting nighttime visual surveys (see below) were equipped with lists of snakes under study; where possible, snakes were scanned for PIT tag identification prior to capture and left untouched if on the list. Where this was not possible, snakes that were captured then determined to be under study were immediately released without further measurements.

**Figure 2:**
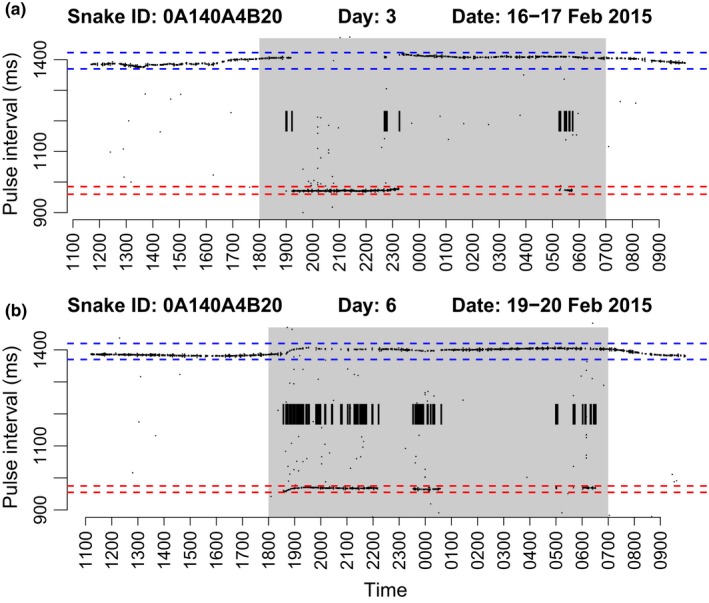
Nightly activity plots from the same snake during low (a) and high (b) activity periods. Shaded gray areas indicate hours of darkness from 1800 to 0700. Blue and red dashed lines represent ranges of pulse interval states. Black lines at *y* = 1200 indicate state transitions (“tips” of the transmitter) identified by the postprocessing algorithm

Automated receiver downloads were processed to extract interpulse interval data. We created a custom script in R (R Core Team 2015) to classify pulse intervals to one of two tipping states while ignoring noise. A state transition between pulse interval rates was recorded as a transmitter “tipping event.” The count of state transitions (“tips”) per hour was recorded as the response variable indicating relative activity levels for statistical analysis. State transitions that lasted for only one pulse interval were ignored as noise, and any recording period that did not include at least thirty pulse intervals was ignored as having insufficient data; these determinations were somewhat arbitrary, but would introduce no bias between treatment groups. Examples of nightly pulse intervals and state transition recordings are graphically represented in Figure 2.

Hourly tip counts, as a proxy for snake activity level, were modeled as a negative binomial response variable in a mixed‐effects generalized linear regression (R package “glmmADMB”). Because time series data are not temporally independent, the tip counts from the previous hour were included as a predictor variable in an autoregressive (AR1) model, of the general form:

$$y_{t}=\beta_{0}+\beta_{1}y_{t-1}+\beta_{2\ldots n}+\in_{t},$$

where *β*_1_ *y*_*t*‐1_ is represented with the “AR1” term. Only *y*‐values where both *y*_*t*_ and *y*_*t*‐1_ met quality control standards were used, and data from *y*_1200–1300_ were used only as the AR1 value for *y*_1300–1400_. The candidate model set was constrained to include only models including AR1 term. *Post hoc* models also considered the significance of variation by treatment day [“DAY,” levels 1–7] or diel period [“DIEL,” levels “day” for hours 0700 to 1700 or “night” for hours 1800 to 0600] as blocking factors and a “MEAL*DAY” or “MEAL*DIEL” interaction terms.

### 2.5 Daily relocation distance

We recorded the general geographic locations of daytime refugia within the snake enclosure on a daily basis by homing to the VHF transmitter signal with a handheld receiver and antenna unit. We identified the location as closely as possible while attempting to minimize disturbance to vegetation in order to not cause spurious snake activity. We obtained estimated location coordinates with handheld global positioning system (GPS) units. The Euclidean distance between successive daily refugia locations was calculated as the daily relocation distance, a standard daily movement metric of brown treesnake telemetry studies (e.g., Anderson et al., 2005; Christy et al., 2017; Santana‐Bendix, 1984; Siers et al., 2016; Tobin et al., 1999), although it is well understood that the actual foraging/movement path can far exceed this distance (Christy et al., 2017; Clark, 1998; Lardner, Savidge, Reed, & Rodda, 2014; Tobin et al., 1999).

Snake movement was constrained within this 5‐hectare enclosure. The enclosure was diamond‐shaped with four sides measuring 220 m each; the maximum interior dimensions were 278 m and 340 m between opposite corners. Although the enclosure limits long‐distance movements, the dimensions were largely adequate to allow expression of the reported average daily movement distances (see Discussion).

Because distributions of daily relocation distances are heavily skewed, with many low values and long right tails, normality was improved by natural log transformation after adding one to all values to prevent “ln (0)” errors. Effects of feeding status, sex, length, and body condition were explored in a linear mixed‐effects model with snake ID as a random effect. *Post hoc* observations indicated that movement lengths were greater on Day 1 for both fed and unfed treatment groups, so additional terms for “DAY” and “DAY*MEAL” were considered.

### 2.6 Microhabitat selection

While locating snakes during the day by homing, we also recorded a short description of the snake's daytime refugium and its estimated height above ground level (m). Refugium descriptions were consolidated into *post hoc* categories for analysis: *Pandanus* spp. (commonly referred to as “screw palms,” palm‐like trees, with spaces among blade‐like axils often used as refugia by brown treesnakes; Tobin et al., 1999; Hetherington, Coupe, Perry, Anderson, & Williams, 2008), *Flagellaria indica* (a woody vine, often forming dense clusters), broadleaf trees (e.g., *Leucaena*,* Triphasia*,* Hibisicus*,* Premna*, and *Morinda* spp.), dead woody vegetation, or ground (grass, litter, or subterranean spaces). All subjective determinations (e.g., refuge height and type, when the snake was not definitively observed) were made by field personnel who were blind to the feeding status of the snake.

Difference in estimated refuge heights between fed and unfed snakes was tested by mixed‐effects linear regression, and differences in use of each refuge type were investigated with separate mixed‐effects logistic regressions.

### 2.7 Detectability by trapping and visual survey

Nightly trapping occurred throughout the duration of our study, using standard brown treesnake traps as described by Rodda, Fritts, et al. (1999). Each trap was baited with a live mouse in a wire mesh protective bait chamber, contained within the body of the trap. Mice were provisioned with paraffinized feed blocks and fresh potato for food and moisture. Traps also contained a length of plastic pipe to provide a refugium for trapped snakes; this reduces the rate of trap escapes (see Rodda, Fritts, et al., 1999). A 13 × 13 grid of traps was established at 16‐m spacing throughout the entire study area (as per Tyrell et al., 2009), with traps checked each morning and snakes identified by PIT tag, measured, and released at the location of trapping.

Time‐ and distance‐constrained visual surveys were conducted on three to five nights per week along established transects by two teams of two searchers equipped with powerful standardized headlamps (Lardner, Savidge, Rodda, Reed, & Yackel Adams, 2009). Detected snakes were hand‐captured, processed as above, and immediately released at the capture location. On each survey night, approximately one‐third of 27 established transects and four forest plot edges were searched. Visual survey details are more fully described in Christy et al. (2010).

Detectability was modeled as a binomial response variable (detected or not detected) in a mixed‐effects logistic regression. Each of the seven nights following release of a fed or unfed snake comprised one (trap only) or two (trap and visual) “trials,” each reflected with a categorical predictor covariate for effort type [“EFFORT” with levels “trap” or “visual”]. The main fixed effect of interest was the feeding status of the snake in the trial [“MEAL,” levels “Y” or “N”]. We also considered fixed effects of other terms and included a random term for snake ID in all models. *Post hoc* models also evaluated the significance of variation by treatment day [“DAY,” levels 1–7] as a blocking factor and a “MEAL*DAY” interaction term.

## 3 RESULTS

We conducted 48 feeding trials from January 19 to April 19, 2015, along with 74 unfed (transmitter‐only) control trials; control trials exceeded feeding trials to account for the fact that unfed snakes shed transmitters after an average of 4.4 days, while the majority of fed snakes retained transmitters for the full 7‐day trial. Sex ratios, size distributions, and body conditions of snakes in study groups were similar (Table 1). Because most brown treesnakes tend to become reproductively mature between 910 and 1,025 mm SVL (females) or 940 and 1,030 mm SVL (males) (Savidge, Qualls, & Rodda, 2007), this sample included snakes of both prereproductive and reproductive size classes.

**Table 1:** Characteristics of brown treesnakes in study groups

Study group	Sex ratio F:M	Snout‐vent length (SVL; mm) Mean ± *SD* (Range)	Body condition index (CI) Mean ± *SD* (Range)
Treatment (fed large meal)	27:21	1059 ± 110 (808–1256)	−0.024 ± 0.121 (−0.269 to 0.212)
Control (transmitter‐only)	39:35	1043 ± 120 (836–1404)	0.015 ± 0.105 (−0.190 to 0.342)

### 3.1 Hourly activity patterns

**Figure 3:**
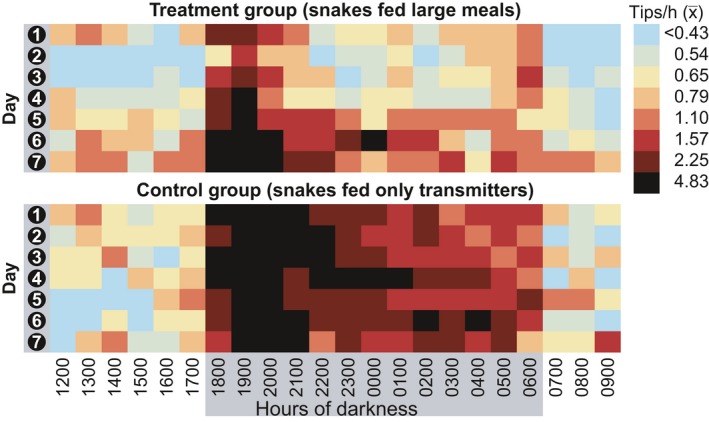
Hourly mean transmitter tipping rates over the seven days following the beginning of trials

We obtained 12,227 hr of automated telemetry activity data meeting quality control standards. Visual examination of the raw hourly average transmitter tip rates (Figure 3) reflects a clear pattern of reduced activity in the treatment group compared to the unfed control group, particularly in the first few days following feeding, with the difference subsiding around Days 6 and 7 postfeeding.

In all negative binomial autoregression mixed‐effects models of hourly tip rates, the feeding treatment effect was highly significant (*p* < 0.001). In the AIC_*c*_ model selection process considering the effects of treatment and snake characteristics, the MEAL term was included in models carrying 100% of the summed model weight (*Σw*_MEAL_ = 1.00). The AR1 autoregression term was highly significant (*p* ≪ 0.001) in all models, confirming temporal correlation of tipping counts. The top model included additional effects of snake size (*Σw*_SVL_ = 1.00 and $\Sigma w_{{\mathrm{SVL}}^{2}}=0.61$) and body condition (*Σw*_CI_ = 0.61), although p‐values for these terms were only marginally significant or nonsignificant (*p* = 0.039, 0.080, and 0.080, respectively). This top model accounted for 26.8% of the weights in the model set. Sex was not an important predictor of activity level (*Σw*_SEX_ = 0.28). The top model outperformed the highest ranking model that did not include the MEAL term by −80.60 AIC_*c*_ units, and the ANOVA *p*‐value for comparison of these two models was ≪0.001. The random effect of snake ID was estimated with a variance of 0.2472 and standard deviation of 0.1572 and improved the model fit by −29.2 AIC_*c*_ units over the same model without the random effect (ANOVA *p*‐value for model comparison ≪ 0.001). A negative binomial dispersion parameter estimate of 0.528 (standard error: 0.012) demonstrated that the data set was overdispersed with respect to a simpler Poisson distribution, justifying use of the negative binomial distribution.

*Post hoc* modeling considering the addition of interaction effects of MEAL with hour [HOUR], trial day [DAY], classification of hours into daytime (0700–1800) or nighttime (1800–0700) [term “DIEL”], and a MEAL*DAY*DIEL interaction into the top model indicated that, despite the high number of parameters (48), variability in this large data set supported a MEAL*HOUR interaction at −134.0 AIC_*c*_ units under the next highest model (DIEL*DAY) and −837.4 AIC_*c*_ units less than the tip model without additional temporal effects, for a much better model fit. With additional variation explained by hourly effects, terms for SVL, SVL^2^, and CI changed in significance (*p* = 0.023, 0.062, and 0.170, respectively).

**Figure 4:**
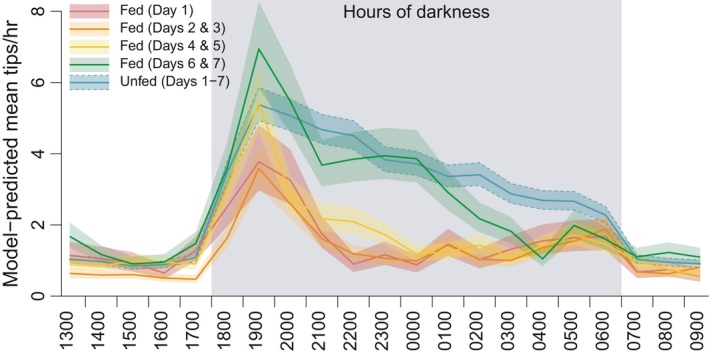
Hourly predicted activity rates based on post hoc pooling of treatment group data by days postfeeding, and pooling all control group days into a single level. Shaded areas represent ± 1 standard error of the estimate

To visualize hourly differences in activity patterns, a *post hoc* classification [“GROUP”] was generated based on the observed differences among days in activity levels for the treatment group and lack of difference among days for the control group; categorical predictor levels were generated for treatment group Day 1, treatment group Days 2 and 3, 4 and 5, and 6 and 7 (pooled), as well as one level for all control group days pooled. Predictions from a fixed‐effects negative binomial model with a GROUP*HOUR interaction term demonstrated depressed hourly activity for the treatment group for Days 1 to 5, with the Days 6 and 7 treatment group activity levels starting to match the untreated group (Figure 4).

**Figure 5:**
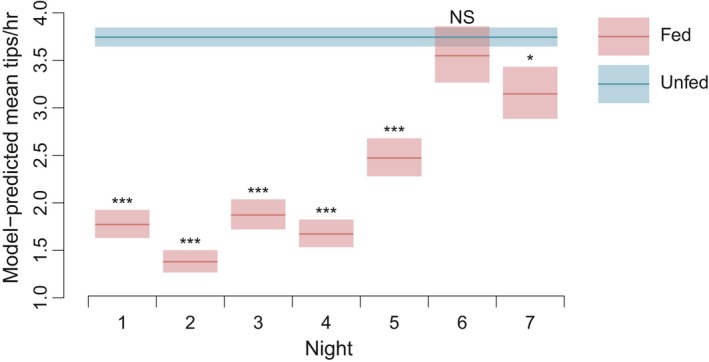
Mean hourly tipping rates by night for treatment (fed) snakes compared to the baseline hourly rate for unfed control snakes (all nights pooled). Shaded areas represent ± 1 standard error of the estimate. “***” *p* < 0.001; “**” *p* < 0.01; “*” *p* < 0.05; “NS” not significant at *α* = 0.05

A simplified fixed‐effects model compared mean hourly tipping rates during hours of darkness (1800–0700) for treatment snakes against rates for unfed control snakes (with all nights pooled into a single estimate). This showed significantly lower activity rates for fed snakes on all nights but the sixth, with a trend toward greater movement at the end of the observation period (Nights 1–5, p ≪0.001; Night 6, p = 0.498; Night 7, p = 0.035; Figure 5).

Official sunset ranged from 1814 at the beginning of field trials to 1834 at the end; this minor variation is not likely to have significant effects on timing of activity within the span of this field study. On Guam, annual cycles in sunset vary by only approximately an hour (1750–1850).

### 3.2 Daily relocation distance

During this study, we recorded 628 daily relocation distances (303 by treatment group snakes and 325 by control group snakes). Mixed‐effects linear regression on log‐transformed daily relocation distances failed to demonstrate an influence of feeding status on daily relocation distance. The top model (DAY + CI + SEX), carrying 57.9% of model weights, did not include MEAL as a predictor, but rather demonstrated influence of DAY (both treatment and control groups had significantly greater average relocation distances on the day of release, *p* < 0.001, *Σw*_DAY_ = 1.0), sex (with males moving greater distances, *p* < 0.001, *Σw*_SEX_ = 0.91), and body condition (higher condition snakes moving further, *p* = 0.019, *Σw*_CI_ = 0.82). *Post hoc* model predictions from fixed‐effects models considering only SEX or CI effects (assuming all other factors at mean values) indicated only modest effect sizes: Males moved 4.13 m per day more than females; snakes at the 90th percentile of body condition moved 2.92 m more than snakes in the 10th percentile (poorer body condition). The MEAL term was included in models carrying only a small proportion of the total model weights (*Σw*_MEAL_ = 0.10). The top model that included the MEAL term (DAY + CI + MEAL + SEX) carried only 5.8% of the model weights, and the estimate for the term was nonsignificant (*p* = 0.484). The mean daily relocation distance for both the fed and unfed treatment groups on the day of release (Day 1) was 32.6 m (*SD* ± 22.7, range 1–107) and for Days 2–6 was 17.8 m (*SD* ± 28.4, range 0–207.5).

### 3.3 Microhabitat selection

**Figure 6:**
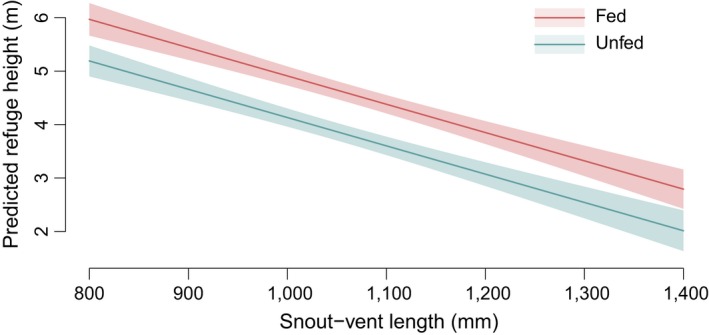
Fed snakes (red) had higher predicted refuge sites (±1 SE) than unfed snakes. Predictions based on the fixed‐effects model. Mixed‐effects significance values: meal effect, *p ≪* 0.001; snout‐vent length effect, *p* = 0.001. Shaded areas represent ± 1 standard error of the estimate

Refugium height was estimated for 504 snake locations (247 fed, 257 unfed). Snakes in the fed treatment group were more likely to choose higher refugia than unfed snakes. The feeding treatment effect [MEAL] in the top model was highly significant (*p* ≪ 0.001), and the effect was included in all top models (*Σw*_MEAL_ = 1.0); the top model, which included the MEAL effect, was 18.28 AIC_*c*_ units lower than the highest ranking model without a MEAL effect. There was a negative effect of snake size (SVL) on refugium height (larger snakes were more likely to take refuge at lower heights, top model *p* = 0.001, *Σw*_SVL_ = 0.97). Only MEAL and SVL were included in the top model, which carried 37.5% of model weights. Two plausible models within 2 AIC_*c*_ units of the top model also carried CI or SEX terms. The top model including CI carried 19.4% of model weights and *Σw*_CI_ = 0.36; the effect of CI was positive (snakes in better body condition were more likely to have higher refugia), but the effect was nonsignificant (*p* = 0.131). The effect of sex (*Σw*_SEX_ = 0.32) was not significant in its top model (*p* = 0.850). There was little support for the SVL quadratic term on SVL ($\Sigma w_{{\mathrm{SVL}}^{2}}=0.14$). Predictions from the fixed‐effects version of the top model reflect only a 0.78‐m increase in refuge height for snakes in the fed treatment group (Figure 6). Exploratory modeling showed no support for a MEAL*SVL interaction.

**Figure 7:**
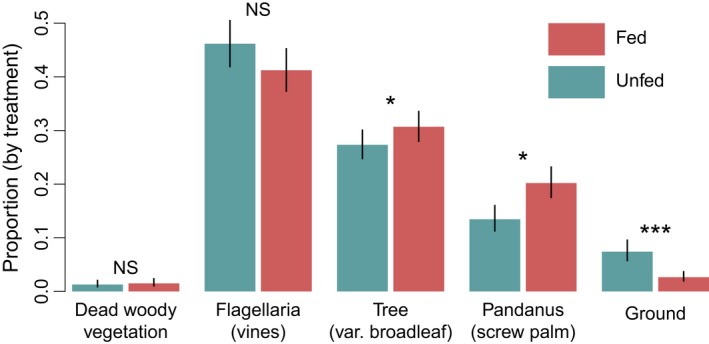
Logistic regression fits and standard errors for differences in proportion of refuge type use between fed and unfed snakes. Fits are from fixed‐effects versions of top mixed‐effects models (constrained to include the MEAL term for sake of comparison). Significance values from top mixed‐effects model (see Table 2). “***” *p* < 0.001; “**” *p* < 0.01; “*” *p* < 0.05; “NS” not significant at *α* = 0.05

Snakes fed large meals differed significantly in their use of some refuge types from unfed snakes, after accounting for influences of snake characteristics (Table 2). While there was no difference in use of dead woody vegetation or *Flagellaria* vines, fed snakes took refuge in broadleaf trees and *Pandanus* screw palms more frequently than unfed snakes (*p* = 0.041 and 0.014) and used refugia on or under the ground less frequently (*p* < 0.001) (Figure 7).

**Table 2:** Top mixed‐effects logistic regression models explaining variation in use of refuge types. All models carried a random effect of snake ID. Sign (+/−) indicate the direction of the term's effect; “Y” and “M” indicate “yes” and “male” for MEAL and SEX categorical predictors, respectively. (NS) = not significant; (.) = *p* ≤ 0.10; (*) = *p* ≤ 0.05; (**) = *p* ≤ 0.01; (***) = *p* ≤ 0.001. Blank cells indicate that the term was not included in the top model. ∆ AIC_c_ is the difference in AIC_c_ between the top models with and without the MEAL term. *Σw*_MEAL_ is the sum of model weights of all models carrying the MEAL term. ANOVA reflects the significance of the difference between the top models with and without the MEAL term compared by a likelihood ratio test

Refuge	MEAL	SEX	CI	SVL	SVL^2^	∆ AIC_*c*_	*Σw*_MEAL_	ANOVA
Dead woody vegetation			+(**)			—	—	—
*Flagellaria* (vines)		M‐(.)	−(***)	+(NS)	− (*)	—	—	—
Tree (various broadleaf)	Y+(*)		+(NS)			1.45	0.70	(.)
*Pandanus* (screw palm)	Y+(*)		+(.)	− (NS)	+(*)	3.41	0.78	(*)
Ground	Y‐(***)	M+(*)	+(*)	+(NS)		16.31	1.00	(***)

### 3.4 Detectability

During 86 nights of trapping throughout the entire study plot and 52 nights of visual surveys searching one‐third of the study plot each night, only two snakes in the feeding treatment group were recaptured; both snakes were hand‐captured after visual detection on the day that they were released after being fed. There were no recaptures of any fed snakes throughout the remainder of the 7 days of each trial. In contrast, there were 11 trap captures and 16 hand captures of unfed control group snakes over the same time period. At 48 × 7 trial nights for fed snakes and 74 × 7 trial nights for unfed snakes, this equals a total capture rate of 5.9 captures per 1,000 trial nights for fed snakes vs. 52.1 for unfed snakes.

**Figure 8:**
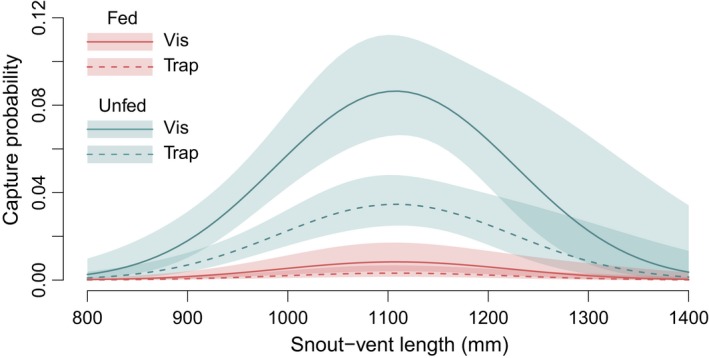
Estimated capture probability (detectability functions) for fed and unfed treatment groups by effort type and snake size, as predicted by fixed‐effects logistic regression. Shaded areas represent ± 1 standard error of the estimate. Note that the detection function for trapping may be depressed by simultaneous visual searching (see Discussion)

In models containing combinations of terms for treatment group, capture effort type (trap vs. visual), and snake characteristics, feeding status was the most important predictor of detection (*Σw*_MEAL_ = 1.00). MEAL was highly significant in all models carrying model weight > 0 (*p* ≪ 0.001). The top model outperformed the highest ranking model without the MEAL term by 17.48 AIC_*c*_ units, and the likelihood ratio test of these two models was highly significant (ANOVA *p* ≪ 0.001). Effort type [EFFORT] also ranked highly in variable importance (*Σw*_EFFORT_ = 0.90). Snout‐vent length was the only other important predictor of capture rates (*Σw*_SVL_ = 0.88, $\Sigma w_{{\mathrm{SVL}}^{2}}=0.71$; Figure 8), with body condition and sex ranking as relatively unimportant (*Σw*_CI_ = 0.28, *Σw*_SEX_ = 0.29). Other plausible models (within 2 AIC_*c*_ units of the top model) varied in the inclusion of either SEX or CI, but when included the terms were nonsignificant (*p* = 0.683 or 0.678, respectively).

## 4 DISCUSSION

Our results indicate that the recent feeding of large meals significantly reduces hourly activity levels and detectability of brown treesnakes and is associated with changes in choice of refugium.

### 4.1 Hourly activity patterns

With the exception of Lardner et al. (2014), the orientation transitions (“tips”) of tip‐sensitive transmitters constitute a previously unobserved metric of snake activity. Coupled with an automated receiver and postprocessing algorithms, to our knowledge, this is the first accounting of the activity patterns of a large number of snakes over the entire activity period for multiple nights (although Tucker et al., 2014 and Tucker, Strickland, Edmond, Delaney, & Ligon, 2015 similarly used automated receivers, interpreting plateaus in fluctuations of signal strength as nesting behavior by box turtles).

Hourly logging of activity levels showed a clear and significant pattern of reduced activity by snakes that had been fed large meals, particularly on Days 1 to 5, with activity levels nearing those of unfed snakes by Days 6 and 7, as indicated by highly influential model terms and effect sizes reflected in Figures 3, 4, 5. This coincides with the observations of Jackson and Perry (2000) that 90% of prey mass ingested by brown treesnakes is digested within 6 days after feeding.

Surprisingly, while snakes fed large meals showed significantly less activity than unfed snakes overall, they exhibited a spike in activity at sunset (1700–1900) approaching the magnitude of that shown by unfed snakes (Figure 4). However, this surge in activity was followed by an almost equally rapid subsidence of activity toward 2200, down to levels roughly equivalent to daytime resting activity rates. This appears to be followed by a slow resumption of modest activity levels, on par with those of unfed snakes, toward 0600, with a similar rapid decline at sunrise (toward 0700). From these data, we cannot definitively assert that recently fed brown treesnakes completely cease foraging, but rather that hourly movement activity is reduced overall. It is plausible to consider that this early evening activity of fed snakes may result from relatively brief searches for more optimal refugia (see “Habitat selection” below). However, we cannot rule out that this apparent activity may indicate reorientation of the body while remaining in the same location.

Rodda, Fritts, et al. (1999) reported the following activity periods for brown treesnakes on Guam: 1800–0200 (active foraging); 0200–1000 (end foraging, location of refugia); and 1000–1800 (resting in refugia). With higher temporal resolution, our results show a slightly different pattern for unfed snakes, with a dramatic increase in activity from 1700 to 1900, a relatively monotonic decline in activity from approximately 1900–0600, and a rapid cessation of remaining activity from 0600 to 0700. However, the general pattern of peak activity between sunset and midnight, tapering off toward sunrise, holds true, as indicated by timing of snake‐caused power outages (Fritts, Scott, & Savidge, 1987; Fritts & Chiszar, 1999), hourly activity levels of 18 juvenile brown treesnakes tracked by Lardner et al. (2014), and Christy et al.'s (2017) observations that brown treesnake displacement distances were three times longer before midnight than after.

### 4.2 Daily relocation distance

Beck (1996) found that fed rattlesnakes (*Crotalus* spp.) moved less (8.5 m/d) than unfed (28.5 m/d; *t* test *p* = 0.06), with exception of one *Crotalus tigris* that traveled 290 m to an overwintering outcrop during the nine days after it fed (this snake had eaten the lowest RPM of the treatment group). Conversely, Blouin‐Demers and Weatherhead (2001) reported that black rat snakes (*Pantherophis obsoleta*) that had been fed a rodent meal were likely to move longer distances than unfed snakes, concluding that snakes traveled further in order to find a refuge with an appropriate thermal environment for digestion (tending to move toward forest edges where basking opportunities were more plentiful).

We hypothesized that brown treesnakes receiving large meals would move less than unfed snakes, due to a postprandial increase in demand to evade predators during this period of increased vulnerability and metabolic commitment to digestion; however, we did not find any differences in daily relocation distance between fed and unfed snakes. We did find that both fed and unfed snakes traveled farther distances, on average, on the first day after feeding or transmitter ingestion, likely as a reaction to being captured and manipulated (including release during the daytime when they are not normally active). Because of strongly skewed distributions, the means of untransformed daily relocation distance values are poor descriptors of movement patterns; however, for the sake of comparison to other data sets, we report a mean relocation distance of 17.8 m for all days after the date of capture and release (median = 8.5, range = 0–207, 25% and 75% quartiles = 3.6 and 26.1). This is considerably lower than mean values reported by Santana‐Bendix (1984) (54.5 m), Tobin et al. (1999) (64.4 m), Anderson (2002) (47.1 m), and Lardner et al. (2014) (43 m for juvenile snakes only). We consider it likely that recording movement distances of a population artificially constrained to a relatively small area (5 ha) over several years may have diminished our ability to detect such a difference, and the lower mean relocation distance of these snakes (less than half of previous estimates) indicates an apparent effect of population bounding on daily movement distances. Further, daily relocation distance is a relatively poor metric of actual nightly movement distances. Brown treesnake foraging patterns are not linear (Rodda, 1992), with nightly cumulative movement distances greatly exceeding daily relocation distances (Christy et al., 2017; Clark, 1998; Tobin et al., 1999); it is possible that unfed snakes moved more (or less) than fed snakes, but selected successive refugia that were roughly the same distance apart as the movement distances of fed snakes. Another alternative hypothesis is that unfed snakes foraged widely between refugia, while fed snakes moved only to locate better refugia to continue crypsis and digestion, as the latter is consistent with the brief activity period of fed snakes just after sunset. Limiting our geographic movement metrics to daily relocation distance within a bound plot is almost certain to diminish our ability to precisely answer these questions; daily relocation data were collected ancillary to the hourly activity data, and these lingering questions could be clarified by an additional study with tracking of free‐ranging snakes throughout the night.

### 4.3 Microhabitat selection

The study of thermal biology is crucial to understanding many aspects of snake ecology (Peterson et al., 1993), and thermal environment is probably the single most proximate factor influencing habitat use by terrestrial reptiles (Blouin‐Demers & Weatherhead, 2001). Most of the attention to postfeeding microhabitat selection by snakes has been regarding postprandial thermophily (PPT): behavioral thermoregulation in which an ectotherm seeks a thermal environment allowing an increase in body temperature (*T*_*b*_) into an optimal range for digestion and metabolism. Snakes benefit from PPT by decreasing the duration of ingestion and improving digestive efficiency (Naulleau, 1983; Peterson et al., 1993; Toledo, Abe, & Andrade, 2003). While frequently observed in laboratory studies across multiple taxa, PPT has infrequently been assessed in the field, and the few studies do not provide a clear picture of a general pattern (Beck, 1996; Blouin‐Demers & Weatherhead, 2001; Hammerson, 1989; Reinert, 1993). Although the benefits of PPT are expected to be greater for snake species that feed less frequently and on larger prey than brown treesnakes, even frequently feeding arboreal green snakes (*Opheodrys aestivus*) have been demonstrated to increase *T*_*b*_ above fasting levels after a small meal (Touzeau & Sievert, 1993); these authors concluded that elevation of *T*_*b*_ several degrees above fasting levels is common in both small and large snakes and appears to not be a function of snake or meal size. Blouin‐Demers and Weatherhead (2001) found that postprandial *E. obsoleta* thermoregulated more carefully than unfed conspecifics. They were more likely to be found basking, tended to use refugia close to forest edges that allowed more basking opportunity, and would relocate over longer distances than unfed snakes to reach such habitats.

Thermoregulation is poorly studied in tropical or nocturnal snakes, but Anderson et al. (2005) observed that brown treesnakes in the laboratory thermoregulated around two distinct *T*_*b*_ ranges (21.3–24.9 and 28.1–31.3°C). In the field, the higher range was only achieved when direct solar radiation was available during the afternoon (when snakes were inactive), and such periods coincided with their only observations of basking behavior. All observed basking behaviors were limited to sightings of exposed loops of coils positioned outside of the confines of refuge sites, in direct sunlight in the afternoon on sunny days. Although it has been suggested that behavioral thermoregulation may be unnecessary in stable tropical climates (Shine & Madsen, 1996), Andersen et al. (*ibid*.) concluded that brown treesnakes do actively thermoregulate.

Forest habitats reduce basking opportunity for snakes, except closer to the canopy where more direct solar radiation is available (Lillywhite & Henderson, 1993), and canopy crowns offer a wider range of temperatures due to solar radiation during the day and radiative cooling at night (Aoki, Yabuki, & Koyama, 1975). The need for direct solar radiation to achieve an elevated *T*_*b*_ in Guam's forests (Anderson et al., 2005) could explain the pattern that brown treesnakes in our study tended to use higher average daytime refuge heights and were rarely observed on the ground. The greater use of *Pandanus* by fed snakes could also be associated with the increased opportunities for exposing body parts to direct solar radiation. In our study, fed snakes did not appear to move more during the day, as might be expected if attempting to thermoregulate in response to changing availability of solar radiation. However, as previously suggested, activity of fed snakes shortly after sundown and lengths of movements between refugia may be due to snakes seeking a better thermal environment for digestion and metabolism during inactive hours for the remainder of the night or in response to changes in temperature, humidity, or air movement between day and night.

Hetherington et al. (2008) found that brown treesnakes in the same general vicinity as our study population (Northwest Field of Andersen Air Force Base) used *Pandanus* crowns for refugia far out of proportion to their availability (3.6% of available cover, but 70% of refuge locations). They speculated that *Pandanus* could provide (a) better protection from predators (with bare trunks leading up to high dense crowns of elongate overlapping leaves with sharp points and barbs); (b) access to preferable microclimates for thermoregulation through ease of basking (Anderson et al., 2005); and (c) pooling of precipitation in axils of crowns allowing maintenance of water balance (water conservation being an important selective pressure for arboreal snakes; Lillywhite & Henderson, 1993).

Costs of avoiding predation can comprise much of an active feeder's foraging costs; small changes in habitat or microhabitat can lead to large changes in exposure to predation (Brown & Kotler, 2004). Lillywhite and Henderson (1993) suggested that arboreal snakes may be more susceptible to predation. With canopy offering less shelter than burrows or crevices, brown treesnakes are often found completely hidden and protected in a variety of elevated microhabitats (Hetherington et al., 2008; Rodda, Fritts, et al., 1999; Tobin et al., 1999). Arboreal position may allow an additional avenue of escape not available to terrestrial animals, namely the ability to rapidly evade capture by dropping from the canopy. This behavior has been frequently observed during hand‐capture attempts by biologists (authors, personal observations).

### 4.4 Detectability

As predicted, our data demonstrate a clear, dramatic, and lamentable decrease in detectability of brown treesnakes following a large meal. Given the current state of Guam's forest fauna, this may be relatively inconsequential, since brown treesnakes have virtually extirpated small birds and mammals from these habitats, leaving little in the way of large prey opportunities outside of urban or savanna habitats (Siers, 2015; Siers, Savidge, & Reed, 2017). However, this postprandial crypsis is particularly troublesome when contemplating the potential for accidental introduction of brown treesnakes to neighboring islands where large prey is abundant (Wiewel, Yackel Adams, & Rodda, 2009). In addition to the demonstrated decrease in effectiveness of trapping where alternative prey is abundant (Gragg et al., 2007), control tools relying on visual detection or active foraging by brown treesnakes (visual surveys, trapping, and toxic baits) or other invasive snakes (e.g., Avery, Humphrey, Keacher, & Bruce, 2014 and Reed, Krysko, Snow, & Rodda, 2010; Reed et al., 2011) may be largely ineffective if snakes rarely forage between periods of inactivity and digestion.

Previous detectability studies in this same closed population (trapping: Tyrell et al., 2009; visual survey: Christy et al., 2010) demonstrated a 7‐day lag in detectability of individual snakes. In both studies, short‐term satiety was indicated as the most plausible hypothesis for this effect. Our observations of depressed activity levels for 5 days after feeding and the 6‐day digestion period observed by Jackson and Perry (2000) are consistent with this hypothesis.

Some of the difference between detection functions for trapping and visual surveys in this study could be a result of the fact that visual surveys were conducted along trap lines on some nights; snakes approaching traps are more visible to searchers, and a snake captured before reaching the trap is not likely to enter that trap on the same night. However, fed and unfed snakes were treated the same in this regard, so no bias with respect to feeding status would be introduced by this effect. The detection functions in Figure 8 should be interpreted with respect to the effect of feeding only and not as an indication of poor trap performance.

### 4.5 Submergent behavior

Brown treesnakes demonstrate reduced activity following ingestion of large meals. This is often speculated to be a general pattern in snakes. Animals that consume a large meal might be more likely to “hole up” and become secretive while digestion occurs (Herzog & Bailey, 1987). Greene (1983) posits that snakes likely use refugia, crypsis, or other defense mechanisms to reduce predation during digestion. Such periods of lowered activity postfeeding may appear as “temporary emigration” in the language of detectability models. However, direct empirical evidence from field experiments, like that presented here, is rare.

Digestion at rest is metabolically demanding and can consume much of the energetic input associated with a successful prey capture (as much as 32%; Secor & Diamond, 1995, 1997). In Burmese pythons, O_2_ consumption can equal that of a sprinting mammal, but sustained for much longer (Secor & Diamond, 1995, 1998). Continuing to forage while under cardiovascular exertion and burdened with ingested prey mass—particularly for an arboreal forager that must defy gravity through greater vertical movements, over discontinuous and unstable substrates—is not likely to be an effective strategy for managing resources that could be allocated to other elements of fitness (growth, healing, reproduction, etc.).

Large meals have been documented to have negative effects on the locomotor abilities of brown treesnakes in laboratory experiments. Crotty and Jayne (2015) found that meal size had a significant negative effect on maximal forward velocity; some brown treesnakes, having taken 1–2 mice, were unable to climb a 45‐degree smooth 24‐mm‐diameter cylindrical rod, which all unfed snakes were able to climb. Sprint speeds were reduced by approximately 50% after taking meals 12% and 21% RPM. Crotty and Jayne (*ibid*.) suggested that slower speeds may be due either to reduced locomotor capacity for speed due to prey weight or to the snakes taking more care in movements to avoid slipping and falling under the altered geometry and balance of the prey bulge. Slips and falls may also be more likely to result in serious physical injury under the increased mass of a substantial prey burden. Under these constraints, the optimal behavior may be to simply remain immobile until the meal has been largely digested or at least entered the small intestine (e.g., Jackson & Perry, 2000; Secor, 2008).

Given the locomotor hindrance of a prey bulge, avoiding predators through crypsis may be far easier than evading them by flight. Brown treesnakes are primarily active foragers (Rodda, 1992), and active foragers have higher rates of encounters with predators (Perry & Pianka, 1997). Brown treesnakes on Guam have been known to be eaten by monitor lizards (*Varanus indicus*) and domestic or feral cats (G. Wiles, unpublished report; authors, unpublished data and personal observations). However, brown treesnakes have no significant predators on Guam (Savidge, 1987), and their dramatic invasion and high densities on this island may in part be due to release from predation experienced in their native habitats. Although predation pressure has likely been relaxed on Guam's brown treesnakes, the relatively short time span over which this population has been isolated may not have been sufficiently long to induce a change in predator avoidance behaviors. We find it reasonable to speculate that brown treesnakes reduce activity and increase crypsis during digestion at least in part to reduce the risk of predation. To the extent that nighttime visual surveyors, intent on finding brown treesnakes, can be considered analogous to nocturnal visual predators, the drastic decrease in detections of snakes that had been fed large meals indicates that postprandial crypsis is effective against visually oriented terrestrial predators.

Our study was not designed to distinguishing among digestion, impaired locomotion, or antipredator behavior as causal mechanisms of postprandial quiescence. Do brown treesnakes decrease activity because of digestive demands or to avoid being preyed upon? Both requirements are likely to have co‐occurred through the evolutionary history of snakes, and it is unnecessary to attribute this behavior to either process alone.

### 4.6 Brown treesnake digestion, meal size, and feeding frequency

Jackson & Perry's description of digestion by brown treesnakes (2000) provided the first detailed account of morphological digestive response in a colubrid. They fed mice averaging 24% RPM to snakes and sacrificed snakes at 1, 3, 6, 14, and 30 days postfeeding. On Day 1, 90% of the prey mass was in the stomach, with 10% transferred to the intestine, and intestinal content peaked on Day 3, at 20% of the mouse's original mass. By Day 6, neither the stomach nor the intestines contained more than 10% of the mouse's original mass, and by Day 14, there were no discernible traces of prey. With respect to SDA, the posterior third of the small intestine showed an increase in mass over time, with the greatest rate of increase on Day 1, demonstrating that brown treesnakes do upregulate digestive capabilities after a meal.

Time spent inactive while digesting cannot be spent on other activities, so foraging behaviors and digestive processes should favor quick but efficient digestion. In general, most arboreal snakes take prey that are not particularly large when compared to other snakes, and the intervals between feeding and defecation are comparatively short (Lillywhite & Henderson, 1993). Brown treesnakes and other colubrids digest 2–3 times faster than typical sit‐and‐wait foragers; brown treesnake intestinal mass peaks at 3 days, while taking 6 days for *C. cerastes* (Jackson & Perry, 2000; Secor, Stein, & Diamond, 1994). At least in some cases, prey mass has relatively little effect on duration of digestion (e.g., *Vipera aspis*, Naulleau, 1983).

Ingestion of a large meal appears to constitute a commitment to a prolonged period of energetic expenditure and vulnerability due to reduced locomotor performance. Brown treesnakes require more time to kill larger prey (Chiszar, 1990). Rodda (1992) reported observing a 1.2‐m brown treesnake on a power line crossbeam seizing a sleeping pigeon or dove by the head (a large prey item, relative to the snake); the bird fell from beam and the snake held on, suspended from the beam, for 22 min before pulling back the dead bird and taking 120 min to swallow it. This likely constitutes a significant expenditure of energy and an extended period of vulnerability. Despite increased susceptibility to predation while encumbered with the mass of large prey, less frequent feeding events on larger meals should ultimately lead to less exposure to predators (Maiorana, 1976).

Based on stomach contents from museum specimens, Greene (1989) reported average RPM for eight species of *Boiga* at 16% (range 0.4%–58%); 13 prey items from brown treesnakes collected in New Guinea ranged from 0.4% to 24% and averaged 10.6%. These could have been partially digested and therefore an underestimate of RPM. More recently, Siers (2015) reported on a comprehensive and habitat‐stratified sampling of brown treesnakes and their stomach contents. Of 1,643 snakes palpated and externally examined for prey bulges, 82.2% exhibited no indication of recent feeding, 14.8% had prey items detectable by palpation only, and 3% had visible prey bulges. Upon necropsy, only 555 (33.8%) contained prey in stomach contents; of those prey items, only 4.7% were ≥10%, 0.91% ≥20%, and 0.36% ≥ 33% RPM. These results indicate that, in recent years, captures of snakes with large prey bulges are exceedingly rare. However, these snakes were captured upon detection by visual survey; our results in this study indicate that visual detection rates for unfed mid‐sized snakes may be as much as 800% higher than that for snakes that had swallowed a large meal within the previous 7 days. A low incidence of observing large prey bulges may be due to a detection bias against recently fed snakes. However, Siers (*ibid*.) also observed that stomach contents from urban and savanna snakes included more large, nonnative prey (commensal birds and rodents), suggesting that, in most forested habitats where larger native prey have been extirpated by brown treesnakes, such large meals truly are a rarity.

## 5 CONCLUSIONS

Brown treesnakes demonstrate changes in activity and microhabitat selection following ingestion of large meals. Activity effects last approximately 5–7 days, a period consistent with digestion (Jackson & Perry, 2000) and previously observed cycles in detectability (Christy et al., 2010; Tyrell et al., 2009). The drastic difference in detectability by trapping and/or visual surveys underscores the importance of preventing accidental introduction of brown treesnakes to other islands (e.g., Saipan, Rota, Tinian, Hawaii) where large prey are abundant. Decreased activity and response to the lures and baits associated with brown treesnake control tools (Clark, Savarie, Shivik, Breck, & Dorr, 2012; Clark et al., 2018; Siers et al., in press) following feeding on large prey items will make eradication of a new incipient population an even more daunting prospect. Strategies for increasing detectability of brown treesnakes in prey‐rich areas may include suppression of large prey (e.g., Christy et al., 2017 and Gragg et al., 2007) to increase the level of foraging behavior, reduce the frequency of submergent behavior, and enhance the relative attractiveness of lures and baits. Cycles of foraging quiescence should also be accounted for in timing of applications of control tools such as aerial delivery of toxic baits (Clark et al., 2018; Dorr, Clark, & Savarie, 2016; Siers et al., in press), to ensure that the availability of baits exceeds the five‐ to seven‐day period during which recently fed snakes may not be foraging.

With respect to fundamental research on animal biology, behavior, and ecology, such evidence of postprandial changes in activity and habitat use is likely to be important for a richer understanding of snake ecology and optimal foraging models for species that consume large meals relative to their body mass during a single feeding.

## DATA ACCESSIBILITY

Data sets analyzed in the study are available through ScienceBase (https://doi.org/10.5066/p9ad7kko).

## COMPETING INTERESTS

We declare that we have no competing interests.

## AUTHOR CONTRIBUTIONS

All authors were engaged in the conception and design of the study. SRS developed the experimental design and study protocol, oversaw data collection, managed data, and conducted analyses. SRS wrote the first draft of the manuscript, and all authors were engaged in critical revision and provided important intellectual content. SRS was employed by U.S. Geological Survey, Fort Collins Science Center, during conception, design, and field activities; funding was provided to USDA National Wildlife Research Center to subsidize data preparation, analysis, interpretation, and reporting. AYA led the concurrent snake detection study that provided data for the detectability component of this study. RNR provided supervision, consultation, and obtained and administered funding. All authors contributed to the final version of the manuscript and approve of all contents.
